# A Human Homozygous *HELQ* Missense Variant Does Not Cause Premature Ovarian Insufficiency in a Mouse Model

**DOI:** 10.3390/genes15030333

**Published:** 2024-03-04

**Authors:** Shabnam Bakhshalizadeh, Anthony D. Bird, Rajini Sreenivasan, Katrina M. Bell, Gorjana Robevska, Jocelyn van den Bergen, Mohammad Asghari-Jafarabadi, Andrew J. Kueh, Philippe Touraine, Anna Lokchine, Sylvie Jaillard, Katie L. Ayers, Dagmar Wilhelm, Andrew H. Sinclair, Elena J. Tucker

**Affiliations:** 1 Murdoch Children’s Research Institute, Royal Children’s Hospital, Melbourne, VIC 3052, Australia; shabnam.bakhshalizad@mcri.edu.au (S.B.); rajini10@gmail.com (R.S.); katrina.bell@mcri.edu.au (K.M.B.); gorjana.robevska@mcri.edu.au (G.R.); jocelyn.vandenbergen@mcri.edu.au (J.v.d.B.); katie.ayers@mcri.edu.au (K.L.A.); andrew.sinclair@mcri.edu.au (A.H.S.); 2 Department of Paediatrics, The University of Melbourne, Melbourne, VIC 3052, Australia; 3 Department of Anatomy & Physiology, The University of Melbourne, Parkville, VIC 3010, Australia; daniel.bird@monash.edu (A.D.B.); dagmar.wilhelm@unimelb.edu.au (D.W.); 4 Hudson Institute of Medical Research, Monash Medical Centre, Melbourne, VIC 3168, Australia; 5 Department of Molecular & Translational Science, Monash University, Melbourne, VIC 3168, Australia; 6 Biostatistics Unit, School of Public Health and Preventative Medicine, Faculty of Medicine, Nursing and Health Sciences, Monash University, Melbourne, VIC 3004, Australia; mohammad.asghari-jafarabadi@monash.edu; 7 Department of Psychiatry, School of Clinical Sciences, Faculty of Medicine, Nursing and Health Sciences, Monash University, Clayton, VIC 3168, Australia; 8 The Walter and Eliza Hall Institute, Parkville, VIC 3052, Australia; kueh@wehi.edu.au; 9 Department of Medical Biology, University of Melbourne, Parkville, VIC 3052, Australia; 10 Department of Endocrinology and Reproductive Medicine, Pitie Salpetriere Hospital, AP-HP, Sorbonne University Medicine, 75013 Paris, France; philippe.touraine@aphp.fr; 11 IRSET (Institut de Recherche en Santé, Environnement et Travail), INSERM/EHESP/Univ Rennes/CHU Rennes–UMR_S 1085, 35000 Rennes, France; anna.lokchine@chu-rennes.fr (A.L.); sylvie.jaillard@chu-rennes.fr (S.J.); 12 CHU Rennes, Service de Cytogénétique et Biologie Cellulaire, 35033 Rennes, France

**Keywords:** premature ovarian insufficiency, HELQ, ovarian development, infertility

## Abstract

Disruption of meiosis and DNA repair genes is associated with female fertility disorders like premature ovarian insufficiency (POI). In this study, we identified a homozygous missense variant in the *HELQ* gene (c.596 A>C; p.Gln199Pro) through whole exome sequencing in a POI patient, a condition associated with disrupted ovarian function and female infertility. HELQ, an enzyme involved in DNA repair, plays a crucial role in repairing DNA cross-links and has been linked to germ cell maintenance, fertility, and tumour suppression in mice. To explore the potential association of the *HELQ* variant with POI, we used CRISPR/Cas9 to create a knock-in mouse model harbouring the equivalent of the human *HELQ* variant identified in the POI patient. Surprisingly, *Helq* knock-in mice showed no discernible phenotype, with fertility levels, histological features, and follicle development similar to wild-type mice. Despite the lack of observable effects in mice, the potential role of HELQ in human fertility, especially in the context of POI, should not be dismissed. Larger studies encompassing diverse ethnic populations and alternative functional approaches will be necessary to further examine the role of HELQ in POI. Our results underscore the potential uncertainties associated with genomic variants and the limitations of in vivo animal modelling.

## 1. Introduction

Premature ovarian insufficiency is a condition that can result from premature depletion of oocytes and/or abnormal ovarian development and affects up to 4% of women of reproductive age. It is characterized by elevated follicle-stimulating hormone (FSH) and amenorrhea before the age of 40 years [1,2]. POI is a leading cause of infertility in women, and while it is believed to have a strong genetic component, its genetic basis is not yet fully understood [3]. Currently, over 80 genes have been linked to POI, with critical roles in various cellular and molecular pathways such as gonadogenesis, folliculogenesis, endocrine signalling, cell growth and division, DNA damage repair, metabolism and autoimmunity [4,5,6]. However, these genes only account for a minority of POI cases.

Oogenesis is a complex process that involves both mitotic and meiotic cellular divisions, leading to the production of a mature oocyte [7]. In humans, oogenesis begins with rapid mitotic cell divisions that give rise to approximately 7 million oogonia, which either commit to a developmental fate or are lost through atresia [8]. After this initial phase, oogonia enter meiosis during prenatal development and undergo a rapid arrest during the diplotene stage of meiotic prophase I and remain dormant until puberty [9,10]. At that point, just before ovulation, luteinising hormone (LH) secretion triggers meiosis resumption. The primordial follicle, which is present during the initial stages of oogenesis, exhibits extreme longevity and features a distinctive structure that makes the nucleus of the oocyte susceptible to DNA damage [11]. Therefore, the proper functioning of the DNA repair system is crucial for maintaining genomic integrity in oocytes. This protects against the development of germline mutations and eliminates oocytes with compromised DNA integrity during oocyte maturation [12,13]. Numerous genes are involved in regulating DNA replication, DNA damage repair, and homologous recombination during meiosis, many of which are necessary for normal ovarian function and female fertility [14,15]. Disruptions to some of these genes, including *MCM8/9*, *REC8*, *HROB*, *NUP107*, *STAG3*, *HFM1*, *POF1B*, *SWI5*, *HELQ*, *MND1*, *PSMC3IP*, *MSH4/5*, *CSB-PGBD3*, and *ATM* (summarised in Table 1), have previously been associated with POI in females. In prophase I of meiosis, homologous recombination (HR) is triggered by DNA double-strand breaks (DSB) and enables the exchange of large sections of the DNA double helix between paired homologous chromosomes. This dynamic pathway is essential for generating genetic diversity and plays a crucial role in repairing damaged DNA [16,17]. One of the important genes that plays a crucial role in various DNA processes such as replication, recombination, and inter-strand crosslink repair is *HELQ* [18]. Previous studies have linked the ortholog of HELQ in Drosophila melanogaster (Mus301 or Spn-C3) and C. elegans (Helq-1 or Hel-3084) to the repair of meiotic double-strand breaks (DSB) and the activation of the meiotic checkpoint [19,20]. In humans, *HELQ* encodes the protein HEL308, which acts as a DNA-dependent ATPase and DNA helicase. It is believed that helicases are critical for strand invasion as they can unwind the D loop, making homologous DNA available for the invading strand. There are two parallel helicase pathways involved in this process. One pathway involves the helicase HELQ, which is associated with ATR and RAD51 paralogs, while the other pathway involves the helicases MCM8 and MCM9, which are recruited by HROB [18,21]. As reported previously, deficiency of Helq leads to reduced germ cell numbers in both male and female mice indicating the critical role of Helq in germ cell maintenance [22,23]. In humans, *HELQ* is expressed in the testes, ovaries, heart, and skeletal muscle [24]. However, its significance in human reproduction remains unclear and further research is necessary to define its role and underlying mechanisms. A meta-analysis of 22 genome-wide association studies conducted on women of European descent has found a correlation between HELQ and the age of natural menopause [25]. In a previous report, no causative variant in *HELQ* was found among Chinese Han women with secondary amenorrhea [26]. However, a recent study on a large cohort of individuals with POI proposed a potential link between *HELQ* variants and the pathogenicity of POI [27]. In this study, we used WES to identify a novel homozygous missense variant in *HELQ* in a POI patient and investigate its potential pathogenicity using mouse modelling. 

## 2. Materials and Methods

### 2.1. Patient’s Clinical Information

The patient was carefully selected based on clinical consultations, with the exclusion of patients who had a known aetiology of POI, such as those who underwent chemotherapy, radiotherapy, gonadotoxic therapy, or ovarian surgery and individuals with *FMR1* premutation or an abnormal karyotype. The patient described here was born to non-consanguineous parents and received a diagnosis of POI at the age of 18 years. She presented with primary amenorrhea, elevated FSH (125 IU/L), and low AMH (0.15 ng/mL). Abdominal ultrasound revealed small ovaries. Further endocrinological assessment indicated the presence of hyperandrogenism with hirsutism and elevated anti-thyroid antibodies in the patient. All procedures were in compliance with the ethical standards of the Human Research Ethics Committee of the Royal Children’s Hospital, Melbourne. Informed consent was obtained from all individual participants included in the study.

### 2.2. General Molecular Techniques

Genomic DNA was extracted from EDTA-blood samples obtained from the patients manually with the NucleoSpin^®^ Blood XL kit (Macherey-Nagel, Düren, Germany) or using an automated system, Hamilton Microlab STAR and Nucleospin^®^ Blood L kit (Macherey-Nagel, Düren, Germany), following the manufacturer’s instructions. The quality of the DNA was evaluated using a NanoDrop™ 1000 spectrophotometer and the Qubit dsDNA BR Assay (Thermo Fisher Scientific, Waltham, MA, USA). Selected variants were validated by Sanger sequencing using BigDye v3.1 Terminators (Applied Biosystems, Austin, TX, USA) and ABI 3130X (Applied Biosystems, Austin, TX, USA).

### 2.3. Whole-Exome Sequencing 

The genomic DNA underwent WES at the Victorian Clinical Genetics Service (VCGS), with exome capture using SureSelect Human All Exon V6 (Agilent, Melbourne, Australia). The sequencing was carried out on the NextSeq 500/550 (Illumina, San Diego, CA, USA). WES data were processed using a Cpipe pipeline [76] and analysed using SeqR (https://seqr.broadinstitute.org/, seqr v1.0-83775191; accessed on 12 May 2023). Two different approaches were employed to analyse WES data as previously described [62]. The first was gene-centric and focused on gene priority using POI candidate genes (adapted from [62]) and the second was variant-centric and focused on variant priority. For variant-centric analysis, moderate-high priority (MAF < 0.001) biallelic variants in any gene or high priority loss of function variants (MAF < 0.0001) were considered. GnomAD (https://gnomad.broadinstitute.org/, accessed on 12 May 2023) was used for analysing the minor allele frequency (MAF) as well as the tolerance of genes to missense and/or loss-of-function variation. To predict the pathogenicity of variants in silico, several online algorithms were utilised, all of which were accessed on 12 May 2023, including SIFT/Provean (https://provean.jcvi.org/version 2023), Polyphen2 (https://genetics.bwh.harvard.edu/pph2; v2), Mutation Taster (https://www.mutationtaster.org/; v2) and CADD (Combined Annotation-Dependent Depletion) score (https://cadd.gs.washington.edu/snv; v1.6). The conservation of affected residues was analysed using Multiz Alignments of 100 vertebrates (UCSC Genome Browser https://genome.ucsc.edu/; v 2023) as well as the Decipher database (https://www.deciphergenomics.org/; v11.22).

### 2.4. Mice

All procedures involving animals for the generation of knock-in mice were conducted at the Melbourne Advanced Genome Editing Centre (MAGEC) gene editing facility, located within the Walter and Eliza Hall Institute. F1 heterozygous mice of the C57BL/6J background were bred at the Disease Model Unit animal facility of MCRI to establish the knock-in line. Mice were kept in a temperature-controlled facility (25 ± 1 °C) and on a 12 h light: 12 h darkness regimen under specific pathogen-free conditions with mouse chow freely available. The experimental protocols were approved by the Animal Ethics Committee (AEC) of the Royal Children’s Hospital. Cervical dislocation was used as the method of euthanasia for the mice. Testes and ovaries were individually measured and weighed.

### 2.5. Production of CRISPR/Cas9 Knock-In Mice

To generate the *Helq* knock-in mice, a Q161P mutation was introduced within the *Helq* gene located on chromosome 5 of the mice from a C57BL/6J background. 20 ng μL^−1^ of *Cas9* mRNA, 10 ng μL^−1^ of a single guide RNA (sgRNA) sequence (*AAGTATAAAATTTGAGAAGA*) and 40 ng μL^−1^ of the oligo donor (*CTAGATGTCCCTGCTGAACCAGAACCTGGAAGCGATCTTTCGTTTGATGTGCCTTCTTCTctATTTTATACTTTGAAAATCCGCAGAACTCACCAGAAGCTTTGGGCGATCCGTGCACTAG*) (in which uppercase bases denote exons and lowercase bases denote intron sequences) were injected into the cytoplasm of fertilised one-cell stage embryos generated from wild-type C57BL/6J breeders. After a period of 24 h, two-cell stage embryos were transferred into the uteri of pseudo-pregnant female mice. To confirm the integration of the oligo donor, the viable offspring were genotyped by next-generation sequencing using the forward primer *AACCTCACTGACCCGGAAAC* and the reverse primer *ACTCTGAAGCAGCTCTGACG*. Out of the offspring, five F0 mice were selected for subsequent backcrossing with wild-type mice to produce F1 heterozygous mice.

### 2.6. Genotyping

An assay utilizing restriction fragment length polymorphism (RFLP) was used to genotype the *Helq* knock-in mice. The procedure involved amplifying a specific DNA fragment encompassing the *Helq* variant through PCR, followed by digestion with ApoI, an enzyme that selectively cleaves the wild-type DNA but not the DNA carrying the variant. The resulting DNA fragments were then separated and visualized on an agarose gel, enabling the identification of different genotypes (homozygous, heterozygous and wild-type). Restriction digest of the wild-type PCR product resulted in two fragments measuring 296 bp and 404 bp, whereas restriction digest of the *Helq* variant led to a single 700 bp product. The primer sequences used for genotyping were *GCGAAGAGGACATGTTTGGT* as the reverse primer and *CCATATAGCTTTTTGATTCCTTTCA* as the forward primer. Additionally, PCR was performed for the biological sex of the mice. The presence of PCR products for both *Sry* and *Hprt* indicated male sex, while only *Hprt* amplicons were indicative of female sex. We allocated the mice to four different experimental groups: i. Fertility assessment, ii. Histological and morphological analysis of gonads, iii. Quantitative assessment of ovarian follicles, iv. Longevity study for survival/tumorigenesis analysis.

### 2.7. Fertility Assessment

For fertility tests of *Helq*^KI/KI^ knock-in mice, we established five parallel crosses over a period of 6 months. These included a total of 60 mice, divided into six breeding pairs for each of the five different genotype crosses, namely Hom × Hom, Wt × Wt, Het × Het, Hom (male) × Het (female), and Hom (female) × Het (male). Subsequently, the variations in the number of litters and pups among these five breeding groups were evaluated.

### 2.8. Histology and Immunohistochemistry and Reagents

Ovaries and testes were collected from all genotypes of C57BL/6J mice per experimental group (*n* = 72, six mice per sex and per genotype at two time points, 3-month-old and 6-month-old mice). Gonads were fixed (ovaries in 4% PFA, one of the testes in Bouin’s solution and the other in 4% PFA), paraffin-embedded, and sectioned at 5 μm. Every fifth section was collected for morphological, histological, and immunofluorescent analysis, using standard procedures. To illustrate the morphological difference between wild-type and *Helq*^KI/KI^ mice, fixed gonads of mice were sectioned and deparaffinised in xylene and rehydrated in a graded series of ethanol. Hematoxylin and eosin (H&E) and periodic acid-schiff (PAS) were used to stain the prepared sections from both ovaries and testes.

The subsequent primary antibody employed for the immunostaining of the ovarian sections was Rabbit anti-FOXL2 [77] as a marker of granulosa cells of ovaries. Goat anti-rabbit-HRP was used as a secondary antibody. Slides were developed in DAB substrate and counterstained with Hematoxylin.

### 2.9. Quantification of Ovarian Follicular Numbers

Quantification analysis of ovarian sections was performed to determine any differences between the number of follicles in the ovaries of *Helq* knock-in and wild-type mice. Serial sectioning at 5 μm of ovaries from in 6-month-old females (*n* = 21, seven mice per genotype) was performed at Melbourne Histology Platform of the University of Melbourne with every 10th section used for the quantification. 

Immunohistochemistry (IHC) for FOXL2 was conducted to visualise distinct developmental stages of follicles, with a particular focus on identifying smaller primordial follicles. Follicles were classified as primordial if they contained an oocyte surrounded by a partial or complete layer of squamous granulosa cells. Primary follicles showed a single layer of cuboidal granulosa cells. Follicles were classed as secondary if they possessed more than one layer of granulosa cells with no visible antrum. Early antral follicles possessed generally only one or two small areas of follicular fluid (antrum) whilst antral follicles possessed a single large antral space. The follicle classes were quantified separately and using light microscopy. Only follicles with visible oocyte and *zona pellucida* were counted. 

### 2.10. Statistical Analysis

For evaluating the statistical significance of differences between groups we used one-way analysis of variance (ANOVA) followed by Tukey post hoc tests. A *p* value less than 0.05 was considered statistically significant. All analyses were performed using GraphPad (version 9.0.0 for Windows, GraphPad Software, San Diego, CA USA, www.graphpad.com, accessed on 12 May 2023).

## 3. Results

### 3.1. Identification of a Homozygous Missense Variant in HELQ

**Figure 1:**
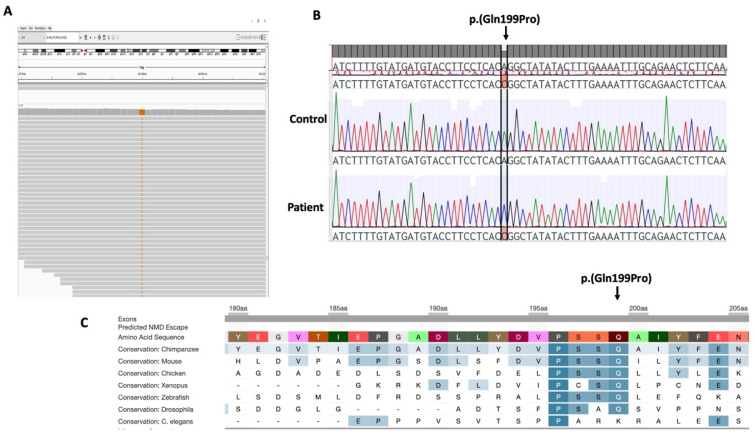
A homozygous missense variant in *HELQ*. (**A**) IGV view of *HELQ* variant (c.596 A>C; p.(Gln199Pro)) variant in an isolated POI patient. (**B**) Sanger sequencing of the patient was consistent with the WES results. (**C**) Decipher view of the variant indicating conservation of the affected residue among the different species.

In an effort to understand the genetic basis of POI, we used WES to analyse a patient diagnosed with POI and primary amenorrhea. In the patient, gene-centric and variant centric analysis detected 41 variants of interest in the proband (Table 2). These variants were further analysed for their likely role in causing the patient phenotype and many of them were discarded based on weak in silico evidence for impact and/or only weak association of the gene with potential POI pathogenicity. We considered a homozygous *HELQ* missense variant (Chr4:83453647T>G, NM_133636.5: c.596 A>C; p.(Gln199Pro)) as the top candidate, primarily because it was the sole gene identified by both gene-centric and variant-centric analyses. Sanger sequencing validated the *HELQ* variant in the patient (Figure 1A,B). This variant is absent in population databases (gnomAD, ExAC) and predicted as pathogenic by online algorithms including SIFT (score 0), Polyphen (Score 0.99), CADD (PHRED score 23.9), and MutationTaster (score 1.00). The *HELQ* gene variant affects an evolutionarily conserved residue (Figure 1C) suggesting that changes at this site are likely to be detrimental to protein function. The effects of the *HELQ* missense variant on the protein structure were analysed using HOPE. The variant residue differs in size and hydrophobicity compared to the wild-type residue. The *HELQ* variant, p.(Gln199Pro), introduces a smaller and more hydrophobic residue at this position potentially disrupting interactions, hydrogen bonds, and proper protein folding [78].

### 3.2. Helq^KI/KI^ Mice Are Fertile 

**Figure 2:**
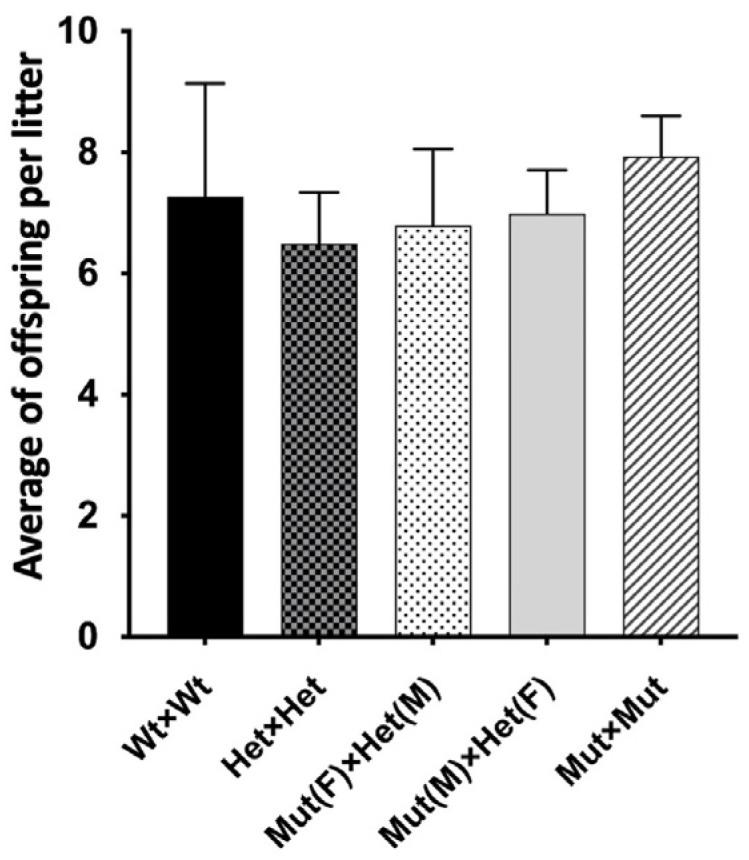
Fertility assessment of *Helq*^KI/KI^ mice. Bar chart shows average offspring per litter in five genotype crosses. No significant differences found in offspring count among five breeding groups over a 6-month breeding period (*n* = 60) (ANOVA *p* > 0.05).

To investigate the effect of the *HELQ* variant we established *Helq* knock-in (*Helq*^KI/KI^) mice using CRISPR/Cas9. Homozygous *Helq*^KI/KI^ mice were born at the expected ratio and had an apparently wild-type phenotype. To assess the reproductive potential of *Helq*^KI/KI^, we established six breeding pairs for each of five different genotype crosses: Hom × Hom, Wt × Wt, Het × Het, Hom (male) × Het (female), and Hom (female) × Het (male). Comparing the number of pups and the interval between litters among these five breeding groups over a period of 6 months showed that there were no significant differences in the numbers of offspring (Figure 2) and the interval between litters among and *Helq*^KI/KI^ and Het/Wt mice. This indicates that both male and female *Helq*^KI/KI^ mice have fertility levels comparable to those of Het/Wt mice.

### 3.3. Helq^KI/KI^ Mice Have Normal Ovarian Weight, Morphology and Follicle Number

**Figure 3:**
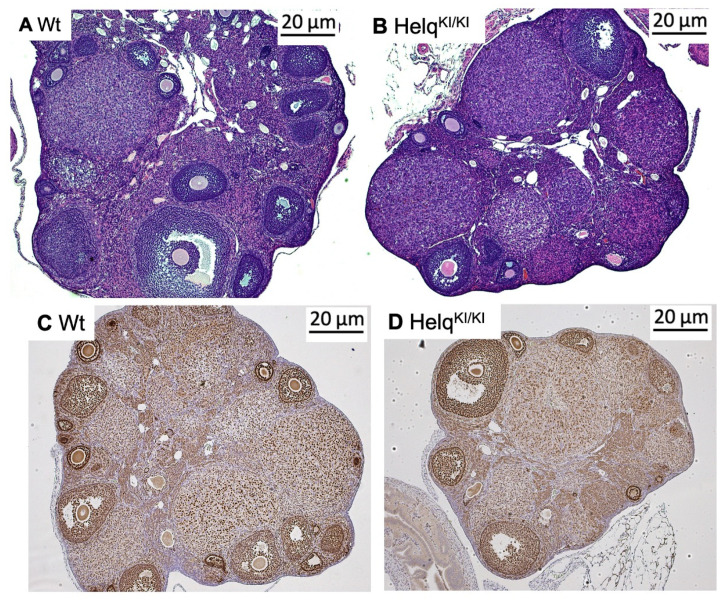
Histological analysis of *Helq*^KI/KI^ ovaries. No differences were observed in the H&E-stained ovarian sections of 3-month-old *Helq*^KI/KI^ mice compared with Wt mice (**A**,**B**). Immunostaining of ovarian sections with FOXL2 showed no differences in morphology of ovarian sections in 6-month-old *Helq*^KI/KI^ mice compared to Het/Wt mice (**C**,**D**) (*n* = 36).

**Figure 4:**
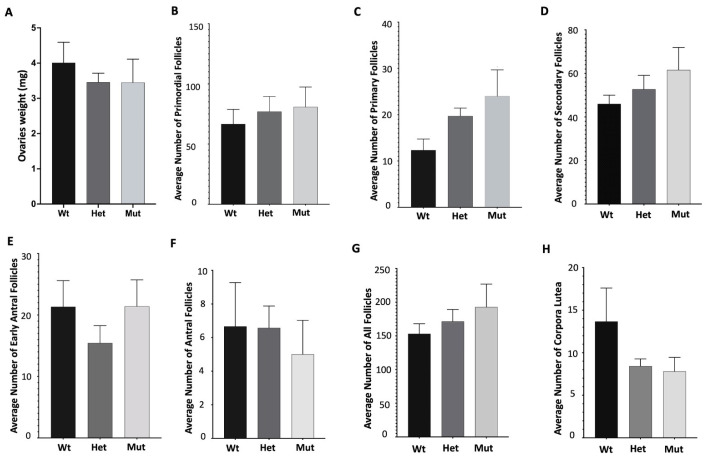
Analysis of follicular numbers in ovarian sections of 6-month-old mice. There was no significant difference in the weights of ovaries of *Helq*^KI/KI^ mice compared to Het and Wt mice (**A**). There were also no significant differences in the average number of primordial (**B**), primary (**C**), secondary (**D**), early antral (**E**) antral follicles (**F**), all follicles (**G**), as well as corpora lutea in *Helq*^KI/KI^ compared to Het and Wt mice (**H**) (*n* = 21) (all ANOVA *p* > 0.05).

In light of the observed dysgenesis and atrophy in ovaries in knock-out mice [22,23], we opted to examine ovarian weight and morphology in knock-in mice. To investigate ovarian morphology between *Helq* knock-in and wild-type mice, ovarian sections from female mice at two time points, 3 months and 6 months, were stained with H&E. Morphological assessment of the H&E-stained sections did not show any apparent differences in *Helq*^KI/KI^ mice compared to the Het and Wt groups (Figure 3A–D). There were no significant differences in ovarian weight of *Helq*^KI/KI^ mice compared to the Het and Wt groups (Figure 4A). Quantification of follicular numbers was performed on ovarian sections obtained from 6-month-old female mice of three different genotypes. The analysis revealed no significant differences in the total number of follicles or individual follicle types between *Helq*^KI/KI^ and Het and Wt ovaries. Additionally, there were no differences in the number of *corpora lutea* (CL) among the mice (Figure 4B–H).

### 3.4. Testicular Weight and Morphology Are Normal in Helq^KI/KI^ Mice

**Figure 5:**
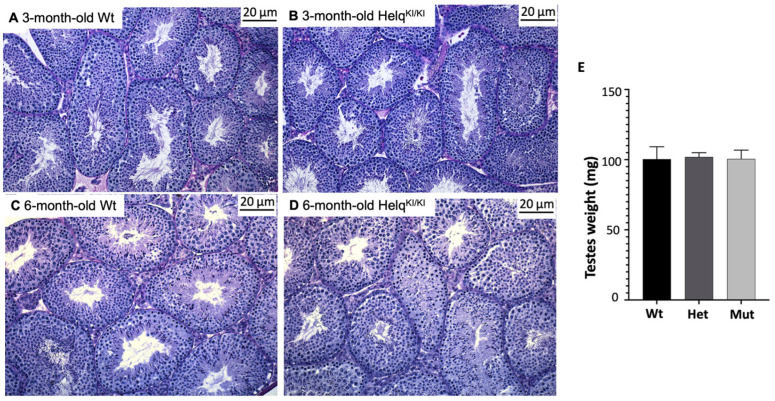
Morphological analysis of *Helq*^KI/KI^ testes. Histological study of PAS-stained testes from 3-month-old (**A**,**B**) and 6-month-old (**C**,**D**) mice did not show any differences between *Helq*^KI/KI^ and Het/Wt mice. There was no statistically significant difference in the weight of testes between *Helq*^KI/KI^ and Het/Wt mice (**E**) (*n* = 36) (ANOVA *p* > 0.05).

Given that *Helq* knock-out mice have smaller testes with regions of atrophied seminiferous tubules [22], we analysed the testicular weight and morphology in knock-in mice. There were no significant differences in the testes weights between the *Helq*^KI/KI^ knock-in and the Het/Wt mice. The testes sections of 3-month-old and 6-month-old male mice were stained with PAS. Morphological assessment results revealed no apparent difference between *Helq*^KI/KI^, het and Wt mice at 3 months and 6 months of age (Figure 5).

### 3.5. Longevity Study for Survival/Tumorigenesis Analysis

Since *Helq* knock-out in mice resulted in increased tumour formation such as ovarian tumours and pituitary tumours [22], we assessed any potential tumour development in *Helq*^KI/KI^ mice. A substantial number of *Helq*^KI/KI^ mice (*n* = 360; 60 mice per sex, per genotype) were assigned to a longevity study group, and their gonads were closely monitored for the development of tumours over a period of twelve months. The results did not reveal any evidence of susceptibility to tumours in the Helq mice.

## 4. Discussion

### 4.1. Missense Variants in HELQ Are a Possible Cause of POI

In the present study, we employed whole-exome sequencing to discover a novel homozygous missense variant in the *HELQ* gene (c.596 A>C; p.Gln199Pro) in an isolated POI patient diagnosed with primary amenorrhea. This variant affects a residue that is highly conserved from archaea to eukaryotes suggesting that changes at this site are likely to be detrimental to protein function. It is predicted to be disease-causing and damaging by multiple in silico algorithms. The *HELQ* gene is a strong candidate POI gene based on studies in mice demonstrating oocyte depletion and infertility in *Helq* knock-out mice. This variant was curated as a Class 3A variant of uncertain significance (VUS) according to modified ACMG criteria [79]. In order to examine the potential implication of this variant on the pathogenicity of POI, additional evidence was sought to establish whether the variant was deleterious. To this end, we generated a CRISPR/Cas9-mediated knock-in mouse model. Despite conservation of this residue and the predicted deleterious nature of the variant by online algorithms, the mouse model harbouring the equivalent of the patient’s *HELQ* variant (p.Gln199Pro) failed to recapitulate the POI phenotype. 

Research using mouse models has provided significant insights into the role of HELQ in male and female reproduction. These studies have shed light on the involvement of HELQ in meiotic processes, such as meiotic double-strand break repair and meiotic checkpoint activation. HELQ has been implicated in germ cell maintenance, ultimately contributing to the regulation of both male and female fertility [22,80]. Previous studies have documented that *Helq* knock-out male mice exhibit hypogonadism associated with a decrease or loss of spermatogonia. Correspondingly, female mice deficient in *Helq* had atrophied ovaries and a diminished number of follicles resembling human POI [22,23]. Multiple lines of evidence have consistently supported a strong association between genes implicated in meiosis and DNA repair and the development of POI [21,81]. However, knowledge regarding the impact of *HELQ* variants in POI patients is based on a limited number of studies. *HELQ* was first identified to be related to age at natural menopause in humans through a comprehensive meta-analysis of 22 genome-wide association studies involving women of European descent. This finding could highlight the potential involvement of HELQ in female reproduction, given that menopause represents the cessation of ovarian function and serves as a reliable indicator of ovarian aging in women [25]. In contrast, a subsequent study failed to identify any plausible causative variants in the *HELQ* after sequencing all 18 exons and exon–intron boundaries of *HELQ* in a cohort of 192 Chinese Han women with POI [26]. Furthermore, two known single-nucleotide polymorphisms (SNPs), rs1494961 and rs2047210, identified within the *HELQ* gene in this study, revealed no significant disparity in allele frequency between patients and controls [26]. A recent study has identified a homozygous truncated variant in exon 17 of *HELQ* (c.3095delA; p.Tyr1032SerfsTer4) using WES in a POI patient who presented with secondary amenorrhea and born to consanguineous Moroccan parents [27]. Chromosomal studies in the patient’s lymphocytes revealed spontaneous chromosomal breaks enhanced by mitomycin. Because only one POI patient has been identified with biallelic variants in *HELQ*, it remains a gene of uncertain significance for POI pathology. Identifying additional POI patients with disruption to this gene will consolidate *HELQ* as a bona fide POI causative gene. Given that the variant in our patient was a Class 3A VUS, we sought additional support by disease modelling to determine its causal role in POI. 

### 4.2. HELQ Missense Variant Remains a Variant of Uncertain Significance

Despite the previous *Helq* knock-out studies, the *Helq* knock-in mice harbouring the *HELQ* variant (p.Gln161Pro equivalent to human, p.Gln199Pro) found in our POI patient does not recapitulate the POI phenotype. Both male and female *Helq*^KI/KI^ mice exhibited fertility levels similar to those of Wt mice. Our assessment of ovarian and testicular tissue through histological analysis revealed no apparent differences between the *Helq*^KI/KI^ and Wt mice. The biallelic nature of the *HELQ* gene variant combined with deleterious in silico predictions and high evolutionary conservation led to this variant being considered of high clinical relevance. However, our mouse model failed to provide the functional support necessary to promote this *HELQ* gene variant to a likely pathogenic status. This could be due to (1) the variant being an incidental finding and unrelated to POI pathogenicity or (2) the failure of the mouse model to functionally mimic the human scenario. Mouse models, while valuable tools for studying human diseases, may not fully mirror the complexity and intricacies of human biology [82,83,84]. Despite their genetic similarity to humans, they often face criticism for their limited ability to accurately recapitulate human disease phenotypes [82]. Furthermore, it is crucial to consider the potential limitations of the knock-in strategy and the specific genetic context of the variant within the mouse genome. The introduction of a single variant in isolation may not fully capture the genetic interactions and regulatory mechanisms present in the human genetic background [85,86]. Mouse strains used in research studies often have different genetic backgrounds, which can influence the manifestation and interpretation of human variants, a factor that is sometimes overlooked. These genetic differences can interact with the introduced variant and produce unpredictable functional consequences of the variant due to the dissimilarities in protein structure, expression patterns, or compensatory mechanisms [82,87,88]. A previous report has highlighted molecular differences within mice of 129 substrains [89]. Similarly, the International Mouse Phenotyping Consortium (IMPC) has shown that all commonly used C57BL/6 strains, in particular the substrains C57BL/6J (used here) and C57BL/6N, are not genetically identical and exhibit phenotypic differences [90].

Furthermore, DNA repair variants appear to exhibit increased variability dependent on the genetic background. For instance, a previous study demonstrated that a mouse knock-in model of DMC1 M200V, harbouring a human infertility allele of the meiotic recombinase DMC1, did not affect fertility in mice [91]. Despite the fact that the DMC1 variant (M200V) was found pathogenic in an African woman with POI [55], both *Dmc1*^M200V/M200V^ female and male mice did not exhibit any abnormalities in their gonads which was similar to the *Helq*^KI/KI^ mice phenotype observed in our study. By contrast, subsequent research indicated a detrimental effect of this DMC1 variant in biochemical assays, and the introduction of the corresponding variant in the *Schizosaccharomyces pombe dmc1* ortholog resulted in a significant decrease in meiotic recombination [92]. This underscores the significance of utilising alternative approaches to validate the variants associated with human infertility. Additionally, given that POI is a heterogeneous disorder with various genetic causes [2], the genetic background of mouse models used to study POI may not fully represent the diverse genetic landscape observed in human patients [39]. Identified POI-related gene variants cannot be completely dismissed or ruled out as there may be intergenic interactions that might influence their effects [15]. 

A recent study highlighted a divergence in the functioning of DMC1 between mice and humans. A pathogenic homozygous frameshift *DMC1* variant (p.Glu10Asnfs*31) was identified in two sisters from a Chinese family who exhibited diminished ovarian reserve with few antral follicles but successful retrieval of metaphase II oocytes from one sister. In contrast, female *Dmc1^−/−^* knock-out mice demonstrated a complete failure of follicle development [93]. Moreover, previous studies in mice showed sexual dimorphism as a consequence of knocking out *Mcm9*, a DNA damage repair gene, in mice. Although the female *Mcm9* knock-out mice were sterile due to a lack of oocytes, male knock-outs were fertile with fewer germ cells compared to their Wt counterparts [94,95]. By contrast, both male and female humans are infertile if the *MCM9* gene is disrupted [30,96]. These studies highlight some of the limitations when using meiosis and DNA repair genes in mouse models when trying to recapitulate gonadal development processes. 

A discrepancy between mouse models and human infertility can result from divergent roles of the affected genes but also from different effects of variant residues in mouse vs human proteins. For example, although *Spata16* knock-out causes infertility in male mice, mice with a knock-in of a variant that segregated with male infertility in a pedigree with three affected individuals [97] retained fertility [98]. It remains possible that the p.Gln161Pro *Helq* variant does not disrupt mouse *Helq* function while the p.Gln199Pro *HELQ* variant may still be deleterious to human HELQ. 

To explore the wider implications of the *Helq* variant on long-term health, specifically regarding cancer predisposition, we monitored *Helq*^KI/KI^ mice for a duration of 12 months. Our primary focus was to monitor for the development of tumours, particularly in the gonad. Unlike *Helq* knock-out mice, the Q161P knock-in mice had no significant tumour development compared to the Wt mice. Although HELQ-deficient human and mouse cells are more sensitive to DNA interstrand crosslinks (ICLs)-inducing agents, suggesting a role for HELQ in the processing of ICLs and tumour suppression [18,22,80], the role of HELQ in cancer development requires further elucidation. For example, screening of 185 Finnish breast or ovarian cancer families for germline variation in the *HELQ* gene showed no likely causative variants. Likewise, studying the association of common variation in the *HELQ* gene with breast and ovarian cancer risk through haplotype analysis did not show any differences between affected cases and healthy population controls [99]. We currently lack a comprehensive understanding of the role of HELQ in cancer development. Notably, neither our genetically modified mice nor the patient under study exhibited signs of cancer. The lack of cancer-related findings in our study does not imply a discrepancy; instead, it underscores the complexity of the involvement of HELQ in cancer, which remains an area requiring further investigation.

### 4.3. Functional Validation Is Important for Human Variant Curation

Curating variants as pathogenic directly without functional/in vivo validation can carry certain risks and limitations. Without disease modelling, the functional consequences of variants may remain uncertain. Computational algorithms play a valuable role in estimating the potential impact of a variant on protein function. However, relying exclusively on these algorithms as an endpoint during the validation process is discouraged due to their limited reliability [100,101]. Therefore, to better understand *HELQ* variant consequences in DNA repair pathways and their relevance to POI pathogenicity, it is crucial to combine insights from mouse models with human studies. Given that only one POI patient has previously been reported with biallelic variants in *HELQ*, it remains a gene variant of uncertain significance (VUS) in terms of potential POI pathogenicity. The identification of additional variants in independent families will strengthen the evidence that *HELQ* gene variants, particularly missense variants, can be causal. These studies could encompass in vitro investigations such as measurement of the stability of variant HELQ or its capacity to support DNA damage repair and the use of patient-derived cells, particularly patient lymphoblasts, which can be tested for their sensitivity to DNA damage. Evidence of an increased sensitivity to DNA damage would support a causal role of the *HELQ* variant. Unfortunately, such cell types were not available in the current study. By integrating various approaches, we can bridge the existing gaps and enhance our understanding of how HELQ deficiency affects DNA damage repair and reproductive dysfunction in humans.

## 5. Conclusions

In conclusion, we have identified a homozygous missense variant in *HELQ* (p.Gln199Pro) in a woman with POI presenting as primary amenorrhea. A *Helq* knock-in mouse model failed to recapitulate the POI phenotype and thus the *HELQ* gene variant remains a VUS. Relying solely on in silico predictions is insufficient to understand the true impact of variants on pathogenicity. Although the *Helq* mouse model failed to support pathogenicity of this novel *HELQ* gene variant, the limitations of mouse models such as incomplete conservation and inability to represent the human genetic background may have masked the phenotypic consequences. Further research employing robust and complementary functional validation approaches including additional patients with independent *HELQ* variants is necessary to establish a more comprehensive understanding of the role of *HELQ* variants in POI and to facilitate patient management.

## Figures and Tables

**Table 1:** Candidate genes involved in meiosis, DNA replication, and repair implicated in premature ovarian insufficiency. AR: Autosomal recessive variants have been reported. AD: Autosomal dominant variants have been reported.

Gene	Inheritance	Reference
*MCM 8*	AR, AD	[21,28,29,30,31,32,33]
*MCM 9*	AR, AD	[30,34,35,36,37,38]
*REC8*	AR, AD	[21,39]
*HROB (C18orf53)*	AR	[21,27]
*NUP107*	AR	[21,40]
*STAG3*	AR	[21,41]
*HFM1*	AR	[21,42]
*POF1B*	AR, AD	[21,43,44]
*SWI5*	AR	[27]
*HELQ*	AD, AR	[25,27,45]
*MND1*	AR	[46]
*PSMC3IP*	AR	[47,48]
*MSH4*	AR	[27,49]
*MSH5*	AR, AD	[50,51]
*MSH6*	-	[45]
*CSB-PGBD3*	AD	[52]
*ATM*	AD	[27]
*PRIM1*	-	[25]
*DMC1*	AR	[53,54,55]
*MEIOB*	AR	[56,57]
*SYCP2L*	AR	[58]
*SYCE1*	AR	[59]
*HSF2BP*	AR	[60,61]
*TP63*	AD	[62,63]
*ZSWIM7*	AR	[64,65]
*FANCM*	AR	[27,66,67]
*FANCL*	AD	[68]
*FANCA*	AD	[69]
*FANCU (XRCC2)*	AR	[70]
*FANCI*	-	[25]
*BRCA1 (FANCS)*	-	[45]
*BRCA2 (FANCD1)*	AR	[27]
*SMC1B*	AD	[39]
*SGO2*	AR	[71]
*SPIDR*	AR	[27,72]
*EXO1*	AD	[25,73]
*RAD51*	AD	[73]
*WDR62*	AD	[74]
*NBN*	AR	[75]
*UIMC1*	-	[25]
*ERCC6*	AD	[27]
*LLGL1*	AD	[45]
*BOD1L1*	AD	[45]

**Table 2:** Genes identified with variants of interest after filtration.

Gene-Centric Analysis	Variant-Centric Analysis (Recessive)	Variant-Centric Analysis (LOF)
Moderate-high priority, MAF < 0.001, high-quality, POI candidate genes	Moderate-high priority, MAF < 0.001, high-quality, recessive inheritance	High priority, MAF < 0.0001, high-quality
14 variants (14 genes): *INSRR*, *LAMC1*, *VWA5B2*, ***HELQ***, *MRPS30*, *BBS9*, *RGS22*, *PCSK5*, *CENPJ*, *MLH3*, *MRPS11*, *SLX4*, *LONP1*, *BMP2*	14 variants (8 genes): *WDR78*, ***HELQ***, *ZBED8*, *ZBTB43*, *DEPDC7*, *CD63*, *LIPC*, *ZNF20*	14 variants (12 genes): *PRAMEF4*, *MROH7*, *ZBTB41*, *OSMR*, *STK32A*, *RABEPK*, *C10orf90*, *SLC6A5*, *LINS1*, *PTPN2*, *ZNF544*, *TSPAN6*

## Data Availability

Some or all datasets generated during and/or analysed during the current study are not publicly available but are available from the corresponding author on reasonable request.
